# Depression and cardiovascular symptoms in a diverse Frontotemporal Dementia cohort, enriched for Caribbean Hispanic patients

**DOI:** 10.1002/alz.092514

**Published:** 2025-01-09

**Authors:** Anisley Martinez, Tianjie Gu, Maryenela Z. Illanes‐Manrique, Sergio J. Tejada, Pedro R. Mena, Heriberto Acosta, Christian Camargo, Xiaoyan Sun, Michelle Marrero Alfonso, Bernard Baumel, Mario Cornejo‐Olivas, Sheila Castro‐Suarez, Jeffery M. Vance, Michael L. Cuccaro, Margaret A Pericak‐Vance, Karen Nuytemans

**Affiliations:** ^1^ John P. Hussman Institute for Human Genomics, University of Miami Miller School of Medicine, Miami, FL USA; ^2^ John P. Hussman Institute for Human Genomics, University of Miami Miller School of Medicine, Miami, FL, USA, Miami, FL USA; ^3^ Neurogenetics Research Center, Instituto Nacional de Ciencias Neurológicas, Lima Peru; ^4^ Carribean Center for the study of memory and cognition, San Juan, PR USA; ^5^ Neurology Department, University of Miami Miller School of Medicine, Miami, FL USA; ^6^ 1501 NW 10th Avenue, Miami, FL USA

## Abstract

**Background:**

Most biomedical data currently available for any disease is derived from studies in non‐Hispanic white (NHW) populations. Specifically, clinical information, genetics as well as biomarkers for frontotemporal dementia (FTD) have been studied predominantly in those NHW populations. For example, a role for depression and cardiovascular symptoms in increased risk for cognitive impairment has been reported; but little information is available on their risk effect in (diverse) FTD.

**Method:**

Our current cohort consists of 135 FTD patients (71% Hispanic, 21% NHW, 6.5% Black/African American), with continuing enrollment from University of Miami (FL), Caribbean Center for the Study of Memory and Cognition (PR) and Instituto Nacional de Ciencias Neurologicas (Peru). All patients were evaluated using NACC Uniform DataSet or equivalent in their preferred language; ∼53% of the cohort also completed the FTD module forms. We compared clinical presentation (age‐at‐onset, ratio behavioral to language variant) in NHW versus Hispanic patients. Additionally, we studied association of Clinical Dementia Rating (CDR) scores with cardiovascular symptoms and depression (Geriatric Depression Score or Cornell Score for Depression).

**Result:**

We did not identify a significant difference in age‐at‐onset, global CDR or ratios of behavioral FTD (bvFTD) versus primary progressive aphasia (PPA) as first symptoms between the NHW and Hispanic patients. We observed association of depression with global CDR≥2, across all patients (p = 0.003) and in Hispanic or Black/African American patients specifically (both p∼0.04). This difference was not driven by bvFTD or PPA subtypes specifically. No significant association was observed for global CDR with diabetes, hypertension or hyperlipidemia in all, Hispanic or NHW patients.

**Conclusion:**

Biomedical research of FTD in underrepresented population groups is necessary as data from research in NHW is not always generalizable across race/ethnicity. Our data suggests that the reported association of depression with higher CDR scores is valid in diverse FTD patient groups; whereas we see less evidence for cardiovascular symptoms being associated with risk for FTD. We are working to expand our efforts characterizing the full clinical and biological presentation of FTD in non‐NHW population groups. The biomedical characterization of FTD across race/ethnicity will help the understanding of disease mechanisms in all patients, ultimately preventing further health disparities.

